# Does Adjuvant Metformin Reduce Olanzapine‐Induced Metabolic Adverse Effects in Patients Diagnosed With Schizophrenia

**DOI:** 10.1002/npr2.70061

**Published:** 2025-09-25

**Authors:** Aquib Butt, Soban Sadiq

**Affiliations:** ^1^ Kent and Medway Medical School University of Kent Canterbury UK

**Keywords:** adjunctive therapy, metabolic adverse effects, metformin, olanzapine, schizophrenia

## Abstract

**Background:**

Olanzapine is an atypical antipsychotic used in the treatment of schizophrenia, bipolar disorder, and major depressive disorder. In comparison to conventional antipsychotics, it demonstrates superiority in binding to serotonin 5HT‐2A than to dopamine D2 receptors, thus presenting as an alternative in having lower extrapyramidal side effects. However, over time, it has become clear that olanzapine carries other prominent adverse effects associated with its use. These relate to the endocrine system and include body weight gain, increased adiposity, insulin resistance, and dyslipidaemia, all of which contribute to metabolic syndrome. Several studies included in this review involved patients with these broader psychiatric diagnoses, not only schizophrenia. This systematic review, therefore, assesses the evidence for the effectiveness of utilizing adjuvant metformin to reduce olanzapine‐induced metabolic adverse effects across these populations.

**Methods:**

Due to the heterogeneity in available data, this systematic review was conducted via a narrative synthesis method. Initially, the search question was formulated utilizing the PICO tool, then a rigorous search strategy was applied to four search engines. Utilizing the PRISMA flow diagram for visualization of the flow of articles, the initial search revealed a total of 71 articles, whereby strict inclusion and exclusion criteria were applied, revealing the final six articles included in the review. Detailed analysis of the articles allowed key themes and outcomes to be drawn upon, including body weight/BMI, waist circumference, glucose/insulin level changes, and lipid profile. This allowed key data to be grouped and narratively analyzed, resulting in the confident formulation of conclusions regarding the ability of metformin to reduce the metabolic adverse effects of olanzapine.

**Results:**

Through this review, adjunctive metformin was shown to play a role in reducing metabolic adverse effects associated with olanzapine. Most notably, its positive effect in reducing weight gain/BMI, triglycerides, liver fat content, and insulin resistance—all of which contribute to metabolic syndrome. Furthermore, the addition of metformin was shown to have no impact on waist circumference and certain lipid parameters such as LDL and total cholesterol, warranting further research. However, employing this evidence in the production of guidelines to benefit patients with schizophrenia remains a challenge due to the lack of evidence in the form of randomized controlled trials surrounding the dose‐dependent effects, as well as age and gender differences of metformin on olanzapine therapy.

**Conclusion:**

Metformin addition to olanzapine therapy showed variable effects on some metabolic parameters such as waist circumference and certain lipid parameters. However, it did show consistent effects in managing body weight/BMI, insulin resistance, triglycerides, and liver fat content. This conforms to previous but limited evidence surrounding the use of metformin in reducing metabolic adverse effects of olanzapine therapy. Based on evidence gaps, this review also proposes areas of additional research and offers recommendations, including the use of longer RCTs, larger demographics to determine if the data can be extrapolated to a wider population, and the use of varying doses to ascertain the dose‐dependent effects of metformin in alleviating metabolic adverse effects associated with olanzapine therapy.

**Trial Registration:**

PROSPERO registration ID: CRD420251015966

## Abstract 2

This systematic review evaluates the efficacy of adjunctive metformin in reducing metabolic adverse effects in patients with schizophrenia treated with olanzapine. Findings indicate metformin consistently improves weight, BMI, insulin resistance, triglycerides, and liver fat content, though effects on waist circumference and cholesterol remain variable, highlighting the need for further trials.

## Graphical abstract

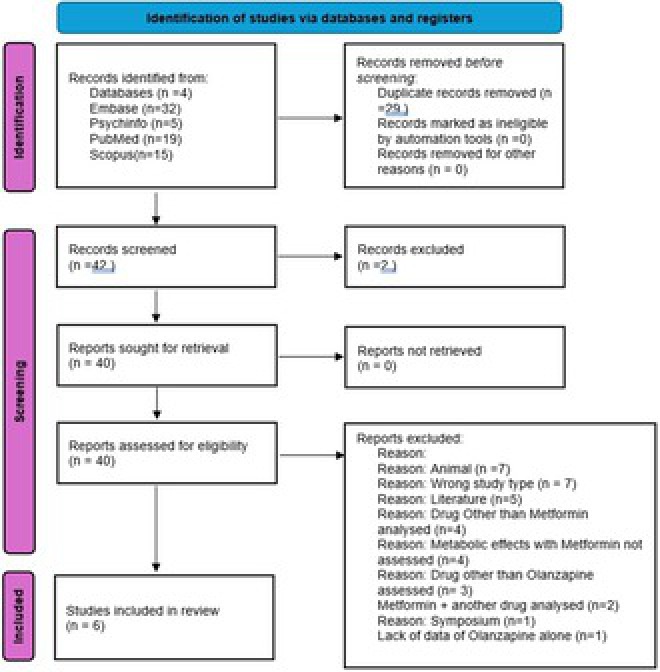

## 1 Introduction

Affecting approximately 1% of the global population, schizophrenia, a psychiatric disorder, was first identified by Beuler in 1908 [1] and is defined by delusions, poor executive function, disorganized speech, and hallucinations [2]. The present understanding of the pathophysiology of schizophrenia includes the dopaminergic hypothesis, denoting alterations in dopamine in the mesolimbic and mesocortical pathways responsible for positive and negative symptoms, respectively [3]. In the 1950s, typical antipsychotics transformed the treatment of schizophrenia, offering effective therapy outside of psychiatric hospitals [4].

Olanzapine, one of the most prescribed atypical antipsychotics, was first approved in 1996 for the treatment of schizophrenia. In comparison to typical antipsychotics, due to its greater affinity for serotonin 2A (5‐HT2A) than for dopamine D2 receptors, it offers clinicians a greater alternative to typical antipsychotics for the treatment of schizophrenia with reduced extrapyramidal side effects (EPSE) [5]. However, a further comparison proved olanzapine to be associated with several metabolic adverse effects, including weight gain, hyperglycaemia, and hyperlipidaemia [6]. Metformin, which has a dual property of enhancing and increasing peripheral utilization of insulin, appeals to negate these effects [7]. Therefore, proving the use of metformin in patients taking olanzapine to reduce its metabolic adverse effects could be pivotal for patients taking olanzapine for schizophrenia [8]. This systematic review therefore assessed the use of metformin in reducing olanzapine‐induced metabolic adverse effects. Due to the large heterogeneity among data, this was conducted as a narrative review [9].

### 1.1 Background Literature Review

#### 1.1.1 Olanzapine

Olanzapine, a thienobenzodiazepine derivative, is a second‐generation (SGA) antipsychotic discovered in efforts to produce alternatives for clozapine without hematological side effects necessitating recurrent blood tests. In its role as one of the most widely used antipsychotics, it is used for the management of schizophrenia, bipolar disorder, and as an adjunct in major depressive disorder [10].

#### 1.1.2 Pharmacology and Metabolic Adverse Effects

Olanzapine, in comparison to conventional antipsychotics, demonstrates a higher affinity for 5‐HT2A than for D2 receptors and has a lower potential to induce EPSE [5]. This represented a significant improvement in managing patients with schizophrenia. However, it has become clear that these atypical antipsychotics carry prominent metabolic adverse effects associated with chronic use, including body weight gain, increased adiposity, insulin resistance, diabetes, and dyslipidaemia. The association of these metabolic sequelae has led to efforts to further characterize and prevent resultant obesity and diabetes [11]. Conforming to these claims, the Clinical Antipsychotic Trials of Intervention Effectiveness (CATIE) Schizophrenia trial, where, among all antipsychotics, olanzapine was associated with the highest risk of weight gain and the highest reason for discontinuation, followed by reduced adherence and resultant relapse and hospitalization [12]. Emphasizing this, Nashed et al. [13], in a study comparing the effects of olanzapine monotherapy with placebo, demonstrated a mean weight gain of 2.1 kg.

#### 1.1.3 Metformin

Metformin, an antidiabetic, serves as a particularly attractive adjuvant in counteracting metabolic adverse effects caused by olanzapine, due to its dual nature of decreasing body weight and improving glycaemic control [14]. A review conducted by Bushe et al. [15] demonstrated that metformin attenuation did not significantly change weight gain in comparison to placebo groups, and more trial data was required to aid in definitive interpretation. However, a study proved to show a weighted mean difference of 5.02 kg lower with metformin in comparison to placebo, conforming to claims that metformin can be used for olanzapine‐induced weight gain [9]. Whilst studies have suggested metformin's ability to negate the metabolic effects of olanzapine, the precise mechanism is unclear. Theories suggesting its role in reducing hypothalamic inflammation [16] and altering gut microbiota lack conclusive evidence [17].

#### 1.1.4 Rationale

Classified by the World Health Organization as a contributor to the global burden of disease, schizophrenia affects 1.5 people out of 10 000 annually [18]. With cases reaching 23.6 million, an increase of 65.85% since 1990, it represents a significant health burden [19]. Olanzapine's superiority over first‐generation antipsychotics towards negative symptoms of schizophrenia [20] makes it particularly attractive; however, coupled with clozapine, it also exhibits the greatest metabolic adverse effects in comparison to all antipsychotics [21]. Due to the varied evidence and gaps in knowledge in previous studies examining the use of metformin for olanzapine‐induced metabolic adverse effects, if adjuvant metformin can be proven to alleviate the metabolic adverse effects of olanzapine, patients with schizophrenia undergoing olanzapine therapy could achieve improved management without the metabolic adverse effects [9].

## 2 Methods

This systematic review was registered in PROSPERO (Registration ID: CRD420251015966). To ascertain relevant information and formulate a research question, the PICO tool was applied (Supporting Information) [22]. The subsequent question was outlined, ‘Does adjuvant Metformin reduce Olanzapine‐induced metabolic adverse effects in patients diagnosed with Schizophrenia?’

### 2.1 Research Aims and Objectives

#### 2.1.1 Aim

- To assess if adjuvant metformin is effective in reducing metabolic adverse effects associated with olanzapine therapy.

#### 2.1.2 Objectives

- To assess through means of a thorough review of literature and clinical trial data the effects of adjuvant metformin on metabolic parameters deranged by olanzapine.
- To identify gaps in current published research regarding metformin use in reducing olanzapine‐induced metabolic adverse effects, to aid in planning future research.

**FIGURE 1:**
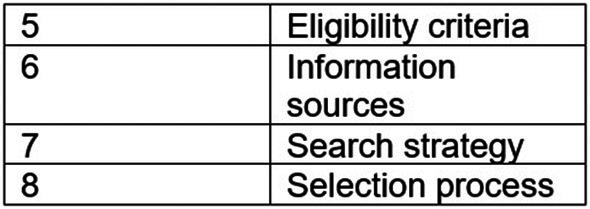
PRISMA‐systematic review steps 5–8.

The objective of a systematic review revolves around a comprehensive and unbiased identification of pertinent studies. Explicit reporting of the search strategy serves as a mechanism to appraise the quality of the search, enabling the reader to judge the credibility and replication of the search when the review is updated [23]. The Preferred Reporting Items and Meta‐analysis tool for systematic reviews methods section was used to develop a search strategy. This section discusses steps 5–8 [24] Figure 1.

### 2.2 Eligibility Criteria

A robust systematic review utilizes keywords, aiding in determining eligibility criteria and allowing indexing systems to produce relevant results [25]. To yield sufficient data to identify and screen relevant studies, keywords from the research question were produced based on PICO analysis (Supporting Information). These included metformin, adjunct, olanzapine, schizophrenia, and metabolic adverse effects. Furthermore, a rigorous inclusion criterion is vital to any systematic review, whereby an inclusion criterion defines key features of the target population used to answer the research question. In contrast, exclusion criteria are defined as features of the participants who could interfere with the success of the study and are thus excluded from the review [26]. These were unique to the study and included participants diagnosed with schizophrenia and taking olanzapine, studies examining metabolic parameters, and excluded studies using examining animal models or using adjuncts other than metformin. The full inclusion and exclusion criteria can be seen in the Supporting Information. Our predefined inclusion criteria and PICO tool specifically targeted patients diagnosed with schizophrenia receiving olanzapine therapy. While some included studies also enrolled participants with other psychiatric diagnoses (e.g., bipolar disorder, schizoaffective disorder), schizophrenia patients were consistently represented and remained the focus of data extraction and synthesis. Therefore, the review question and title remain centered on schizophrenia, consistent with the registered protocol.

### 2.3 Information Sources and Search Strategy

The search was conducted among four databases which formulated the information sources, to which Kent and Medway Medical School (KMMS) granted access; these included Embase, PubMed, Psychinfo, and Scopus, integrating both medical and psychological databases centered around the research question. Boolean operators, including “AND” and “OR”, were also incorporated into the search strategy to search for both terms or either term, respectively [27]. Additionally, truncations and synonyms such as Glucophage for metformin and combination for adjunct were incorporated into the search strategy, allowing a wider search for related terms and preventing errors from failure to retrieve the morphological variant of the keywords in question, allowing a greater number of results to be retrieved [28]. The full search strategy, as well as the number of hits per database, can be seen in the Supporting Information.

### 2.4 Selection Process

**FIGURE 2:**
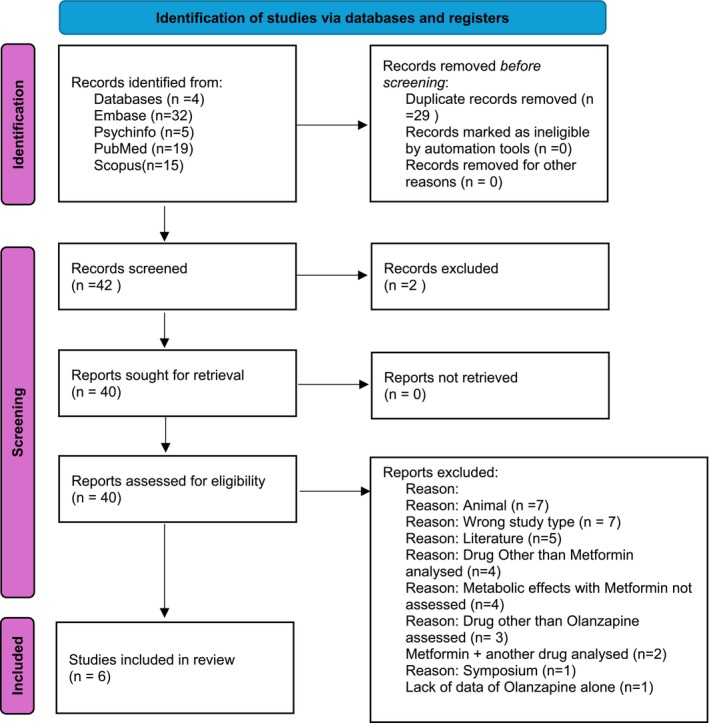
Depiction of the flow of articles through the PRISMA flow chart, to yield the six articles analyzed [29].

Papers were initially screened via their titles and abstracts, with the remaining relevant papers screened for a second time by a single moderator. During the screening process, articles were applied to the inclusion and exclusion criteria, which can be seen in the Supporting Information. Among the primary search, 71 articles were included after the primary search, which went through a review to culminate in the final six articles, as can be seen in the Supporting Information for the table summarizing the final papers in the review. These were applied to the The Critical Appraisal Skills Programme (CASP) tool, Cochrane Risk of Bias for the Randomized Controlled Trials (RCTs) articles 1, 2, 3, 5, 6, and Robin‐I for the open‐label study, article 4. Figure 2 depicts the flow of records through the PRISMA flow and justifications for excluding publications.

**FIGURE 3:**
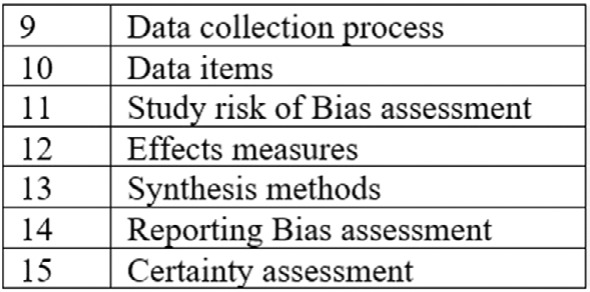
PRISMA‐systematic review steps 9–15.

In line with the PRISMA checklist, the following section discusses steps 9–15, as shown in Figure 3.

### 2.5 Data Collection Process and Data Items

To efficiently and categorically collect and tabulate data, a spreadsheet was employed, with pertinent data related to key characteristics, such as type of study, objectives, metabolic outcomes measured, doses used, and statistical analysis, data items inputted manually, as can be seen in Supporting Information. In addition, another table was formed, which collected data regarding the metabolic outcomes and effect measures of the addition of metformin in the studies, comparing body weight/BMI, waist circumference, glucose/insulin level changes, lipid profile, metabolic outcomes other than the ones specified, and adverse drug reactions; this can be seen in Supporting Information. Data extraction was performed by one author and reviewed by another author.

### 2.6 Study Risk of Bias Assessment

To study the risk of assessment bias, the CASP tool and Cochrane risk of Bias were applied to the randomized controlled trials and Robin‐I for the open‐label study; this can be seen in Supporting Information.

### 2.7 Synthesis Methods

Where it was found that the methodology of each study was diverse, a narrative synthesis was chosen. This provided a textual analysis of the relationships within and between studies as well as an overall assessment of the robustness of the evidence [30]. This was done by first identifying a theory of how the intervention works, why it works, and for whom. Secondly, the selection process of articles was guided by inclusion and exclusion criteria, including date restrictions, obligatory factors related to the research question, and the exclusion of published literature not in English. Once the relevant articles were chosen, a preliminary synthesis of the findings was extracted and tabulated to include key characteristics/descriptives such as the type of study, objective, number of participants, and metabolic outcome measured (Supporting Information). Upon data extraction, it was clear common metabolic outcomes were assessed, allowing the formation of subgroups including body weight/BMI, waist circumference, glucose/insulin level changes, and lipid profile. Data was then grouped and tabulated to explore the relationships within and between studies (Supporting Information) [30]. This allowed for results to be summarized in a consistent narrative format, facilitating focus on identifying trends and themes between evidence to provide a comprehensive summary of the findings [30].

### 2.8 Reporting Bias Assessment and Certainty Assessment

To minimize data loss leading to reporting bias, the research question was formed using the PICO tool. A comprehensive search strategy using multiple databases was used, including Embase, Psychinfo, Pubmed, and Scopus. Coupled with a stringent inclusion and exclusion criteria, this yielded broad yet relevant studies. A pertinent literature search utilizing multiple databases is central to the methodology, and the reliability of the information retrieval process is key to the reliability of a systematic review [31]. In keeping with limiting reporting bias, a PRISMA flow chart was used, detailing how many articles were excluded with relevant reasons (Figure 2).

Certainty assessment was performed on an individual basis; firstly, by assessing each study for its type, sample size, design, appropriateness, results, correlations between metformin and olanzapine‐induced metabolic adverse effects, and overall quality. Secondly, bias tools were employed. The CASP tool (Supporting Information) assessed factors such as the study design, methodology, and result; the Cochrane Risk of Bias (Supporting Information) assessed areas such as the randomization process, deviations from the intended intervention, and measurement of the outcome. Both tools were applied to the RCTs. The ROBINS‐I tool (Supporting Information), which assessed domains such as bias due to confounding, classification of interventions, and selection of participants in the study, was employed for the open‐label study.

## 3 Results

For transparency of evidence quality, attrition and dropout rates were also extracted where reported. Attrition and dropout rates varied across the included studies. The study by Rado and von Ammon Cavanaugh [32] reported one participant dropping out for drowsiness and another for insomnia in the olanzapine/metformin group. The other included studies did not provide specific attrition or dropout rates in the extracted data. Reporting these attrition rates is essential, as they influence both the robustness of the findings and their generalizability to clinical populations.

As shown in the PRISMA flow diagram (Figure 2), 71 papers were retrieved. Entries were removed for several reasons: duplicate entries (29), older than 20 years (2), animal model (7), wrong study type (7), examining a drug other than metformin (4), literature (5), metabolic effects not assessed (4), olanzapine not assessed (3), metformin in combination with another drug assessed (2), lack of data of olanzapine alone (1), and a symposium (1). The final number of studies included in this review was 6: 5 RCTs and 1 open‐label study. Each study was then reviewed, and critical characteristics were extracted and tabulated, as shown in Supporting Information. Upon assessing all papers, it was clear that key outcomes related to metabolic adverse effects could be produced. This included body weight/BMI, waist circumference, glucose/insulin level changes, and lipid profile. One study also assessed the effects of Liver Fat content (LFC), all of which are components related to metabolic syndrome [33].

### 3.1 Body Weight/BMI

In three out of the five studies assessing body weight, the addition of metformin statistically produced a lower increase in BMI and weight (*p* < 0.05) [32, 34, 35]. Chen et al. [36] also demonstrated metformin was able to produce a statistically significant reduction in BMI and weight after 8 weeks of treatment (*p* < 0.01). Wu et al. [34] found that the addition of metformin reduced the number of patients exceeding the threshold for clinically significant weight gain compared with placebo (*p* < 0.001). However, this was not shown by Baptista et al. [35], where there was no difference in in comparison to placebo. One study also studied the effects of the hormone leptin, cortisol, and growth hormone (GH), with the addition of metformin in olanzapine‐treated patients, producing a nonsignificant reduction in leptin when compared to the placebo (*p* = 0.07) and no statistically significant difference between the intervention and placebo group in levels of GH and cortisol [35].

### 3.2 Waist Circumference

Examining waist circumference, results were variable, with three of the studies showing that metformin had no significant difference on the change in waist circumference in comparison to placebo [34, 35, 37]. However, one study showed a greater increase in waist circumference in the olanzapine plus placebo group, compared with the olanzapine plus metformin group, although it was not statistically significant (*p* < 0.085) [32].

### 3.3 Glucose/Insulin Level Changes

It can be noted that, although varied, the addition of metformin did influence glucose and insulin levels. Firstly, insulin resistance, measured via the Homeostatic Model of Insulin Resistance (HOMA‐IR) and insulin resistance index, had a statistically significant (*p* < 0.05) greater increase when olanzapine was combined with placebo compared to with metformin in two studies [32, 34]. Want et al. [38] also supported this, where metformin intervention produced a statistically significant reduction in HOMA‐IR (*p* < 0.05). However, in one of the studies, the difference in change between placebo and metformin was not significant [35]. Secondly, a comparison of fasting insulin revealed that across two of the studies, the addition of metformin produced a statistically significant reduction in insulin (*p* < 0.05) when compared with placebo [34, 37]. Another study further supported this, revealing a statistically significant reduction in fasting insulin after 8 weeks of treatment (*p* < 0.01) [36]. However, this was not reflected by Baptista et al. [35], which showed no significant difference in the comparison of insulin levels between metformin intervention and placebo. Fasting glucose levels showed more equivalent data, with two studies showing that metformin intervention was able to produce a significant reduction in glucose levels [36, 37] and two studies showing that, in comparison to the placebo, there was no statistically significant difference in fasting glucose levels [32, 35]. In studies examining the effects of metformin on HbA1c, albeit reduced, there was no statistically significant difference between the change in HbA1c with the metformin intervention when compared with placebo [32, 38].

### 3.4 Lipid Profile

Results regarding total cholesterol were more varied. Starting with total cholesterol; four out of five of the studies demonstrated no significant difference in total cholesterol levels with the addition of metformin in comparison to placebo. This was also mirrored in the results related to HDL in three of the studies [32, 35, 36, 38]. However, total cholesterol did show a significant reduction with the addition of metformin in one of the studies (*p* = 0.001) [37]. In addition, a comparison of HDL showed that the addition of metformin significantly reduced levels (*p* = 0.007) [35], and in another, it significantly increased levels (*p* = 0.046) [38]. A comparison of LDL showed no significant difference with the addition of metformin. Upon examination of triglycerides, one study showed that triglyceride levels significantly increased after taking metformin (*p* < 0.001) [37], and two studies showed a significant reduction in triglycerides with the addition of metformin (*p* < 0.01) [36], (*p* = 0.041) [38]. One study examining the effects of metformin on liver fat content (LFC) was also able to demonstrate a significant reduction in LFC with the addition of metformin compared with placebo (*p* = 0.009) [38].

A comparison of the level of bias in each study revealed that all studies had a low level of bias.

## 4 Discussion

In interpreting these results, it is important to distinguish between studies examining metformin as a preventive intervention [32, 34, 37], where metformin was initiated alongside olanzapine to mitigate expected weight gain and metabolic changes, versus those that assessed metformin as a therapeutic intervention [35, 36, 38], in which patients had already developed metabolic abnormalities before starting metformin. This distinction is clinically significant, as preventive pharmacological strategies generally require stronger evidence and caution before broad application. The findings of this review should therefore be interpreted in light of this separation.

The key consistent finding of a reduction in weight and BMI gain with adjunctive metformin in patients taking olanzapine could serve to greatly benefit patients. This has also been exemplified in a Cochrane review of pharmacological interventions for the prevention of antipsychotic‐induced weight gain (AIWG), where metformin was the only agent shown to be effective when initiated with an antipsychotic [39]. In addition, metformin treatment has been associated with the most consistent supporting evidence for reducing AIWG. In a meta‐analysis conducted by de Silva et al. [7], metformin treatment resulted in the most significantly better anthropometric parameters compared with placebo. Furthermore, while studies exclusively exploring metformin adjunct therapy on olanzapine therapy are limited, this can be extrapolated in mice models. A study conducted by Suh et al. [16] showed metformin adjuvant therapy was able to significantly reduce weight gain in olanzapine‐administered female mice. While the positive effects of metformin on olanzapine‐induced weight gain have been shown in this study and others, adjuvant metformin use with olanzapine remains infrequent, which could be due to a lack of guidelines or prescribing information [40]. Metformin, in its capacity to be able to lessen AIWG, is related to its ability to counter both hyperphagia and reduced satiety induced by antipsychotics via direct and indirect effects on the gastrointestinal tract, gut microbiome, the CNS, and the gut‐brain axis [41]. Metformin's ability to reduce leptin, shown in this review, further enlightens this. Secreted by white adipose tissue, leptin regulates food intake, metabolism, and energy expenditure via its receptors present in the hypothalamic region of the brain. High leptin levels are directly correlated with obesity and subsequent development of metabolic disease consequences, including insulin resistance, type 2 diabetes, and cardiovascular disease [42]. An increased prevalence of metabolic adverse effects in patients with schizophrenia contributes to lower life expectancy and increased rates of mortality; thus, the use of metformin to prevent weight gain and decrease leptin levels can be beneficial in reducing these outcomes and improving the quality of life for those taking olanzapine.

Assessing metformin's impact on waist circumference, there was no consistent positive change. Impending alterations in waist circumference could require a longer duration to manifest. This is supported by the only study in this review that did report a significant waist circumference reduction [32] and had a longer study duration of 24 weeks in comparison to the others where the maximum duration was 14 weeks. In addition, this was also the only study to use extended‐release metformin, which in other studies has shown to be superior to immediate‐release metformin in tolerability and compliance [43]. This is a particular point of interest for future guidelines, as medication non‐compliance is as high as 40%–50% in patients with Schizophrenia [44].

This review demonstrated the positive effects of adjunctive metformin on insulin resistance, demonstrated by a reduction in the insulin resistance index or HOMA‐IR, which serves as a proven vigorous tool for the surrogate assessment of insulin resistance [45]. This is a particularly significant finding as olanzapine, out of all antipsychotics, is known to have one of the highest risks of new‐onset diabetes, contributing to metabolic syndrome [46]. This finding can be owing to its mode of action; several studies have demonstrated metformin's ability to improve insulin sensitivity can be related to an increased insulin receptor tyrosine kinase activity, enhanced glycogen synthesis, and an increase in the recruitment and activity of glucose transporter type 4 (GLUT4) [47]. In addition, the increased expression and activity of GLUT4 lead to an increased uptake of glucose into cells, achieving glucose homeostasis [38]. However, where it was shown that changes in glucose with the addition of metformin are insignificant, it could have been attributed to a disparity in baseline metabolic status of the patients in this paper, adherence, and age disparity across all the studies. Finally, this study also demonstrated insignificant changes to HbA1c with the addition of metformin, which is a form of glycated hemoglobin and an indicator for blood glucose in the preceding 2–3 months [48]. This suggests that, despite metformin, the metabolic consequences of olanzapine can outweigh the impact of metformin. Unexpectedly, one study also showed a significant increase in HbA1c levels when olanzapine was combined with metformin. Further detailing the study, this could be related to a significant limitation in the study: a variation in baseline BMI and prior weight gain in the participants [35].

Lastly, the heterogeneity of results surrounding metformin's role in lipid abnormalities requires further assessment. Rado and von Ammon Cavanaugh [32] showed an increase in LDL with metformin therapy; however, this could be attributed to LDL not being able to be calculated for two patients in the olanzapine and placebo group due to elevated triglycerides, and thus, they were removed from the analysis. Where the results of Baptista et al. [37] suggested that metformin increased both triglycerides and total cholesterol, this was contrasted by Chen et al. [36] and Wang et al. [38], who were able to prove a significant reduction in triglyceride levels with the addition of metformin. HDL cholesterol, on the other hand, was able to provide a more robust trend whereby Wang et al. and Baptista et al. [37, 38] showed that the addition of metformin significantly increased HDL levels. This is consistent with previous studies where metformin has been proven to reduce antipsychotic‐induced dyslipidaemia, particularly the parameters of total cholesterol and triglycerides, and to increase HDL [49]. Assessing HDL further, it can be shown by Liu et al. [50] that the incidence of metabolic syndrome increases in parallel with a decrease in HDL, which is a positive finding for patients taking olanzapine. The contrary results in HDL shown by Baptista et al. [38] point to a significant limitation of the study mentioned previously related to heterogeneities in basal BMI and previous body weight gain. The addition of metformin also showed to produce a reduction in liver fat content (LFC) [38]. Where 95% of liver fat content exists in the form of triglycerides, and a positive correlation was revealed between decreasing LFC and decreasing triglycerides. A previous study conducted by the same authors revealed that in the acute phases of olanzapine therapy, patients had an increased LFC and fatty liver. This serves as a positive finding, as an increase in liver fat has an originating consequence on metabolic disorders, increasing the risk of both diabetes and cardiovascular disease [38].

Overall, the results of this review serve as a positive outlook for adjuvant metformin in alleviating the metabolic adverse effects of olanzapine and the research question “Does adjuvant metformin reduce olanzapine‐induced metabolic adverse effects in patients diagnosed with Schizophrenia”. Through the effective reductions of parameters related to metabolic adverse effects, metformin acts as a promising candidate not only in improving overall health outcomes but also adherence via a reduction in concerns related to weight gain and metabolic adverse effects. Additionally, while metformin was effective, it is also associated with adverse effects such as gastrointestinal intolerance, vitamin B12 deficiency, headache, and fatigue, which should be considered in clinical application.

### 4.1 Limitations

#### 4.1.1 Demographics and Drug Doses

There were large variations in age, ethnicity, and gender. This introduced a potential for bias if one gender or ethnicity is over or under‐represented. Furthermore, we cannot ascertain if the results can be applied to a wider general population. Likewise, drug doses of both metformin and olanzapine, as well as the length of prior olanzapine therapy, significantly varied throughout the studies. Dosing variations acted as confounding variables, masking the factual relationship between metformin and metabolic effects, making it challenging to conclude key relationships.

#### 4.1.2 Sample Size

The variation in sample size ranged from 24 to 80 between the papers. Importantly, this has a role in impacting statistical power, where studies with a smaller sample size compared with a larger sample size lack sufficient statistical power to be able to detect consequential results. Studies with a larger study size can be able to reduce the impact of random error, whereas smaller population sizes can potentially introduce greater variability in their results due to chance. This also makes it difficult to determine if results can be generalized to a larger population, whereas data sets with a smaller population size can introduce bias by focusing on a narrowly defined population.

#### 4.1.3 Study Design and Methodology

This was carried out as a narrative review due to heterogenicity in results, in comparison to meta‐analysis, which lacks statistical robustness. In addition, the durations of each study also varied significantly, with the shortest duration being 8 weeks and the longest being 24 weeks [32]. This duration bias influenced results by introducing a time‐dependent effect whereby some metabolic parameters such as HbA1c and lipid profile can take time to manifest, and shorter duration studies would miss this; the same applies for ADRs, which may only manifest in longer study durations. Addressing controls for participants, these varied between studies, acting as confounding variables that can impact metabolic outcomes in different ways. Whilst methods across studies varied, one study did not employ a placebo‐controlled group, preventing the elimination of participants' motivation to lower their body weight and change their lifestyle [36].

Lastly, although all patients recruited were taking olanzapine monotherapy, studies by Rado and Cavanaugh, and Baptista et al. [32, 35, 37] recruited patients with other psychiatric diagnoses such as bipolar disorder, schizoaffective disorder, and major depression with psychotic features in addition to recruiting patients with schizophrenia. The studies did not define specific results for each condition; thus, results specific to patients with schizophrenia could not be extracted and were included in the review. Although the title and inclusion criteria were designed around schizophrenia, several of the included studies recruited mixed psychiatric populations. Because schizophrenia‐specific outcomes were not always reported separately, this introduces a potential limitation in the generalizability of findings. Nonetheless, the systematic review remains faithful to its original scope and protocol, which were explicitly focused on schizophrenia.

## 5 Recommendations

In future studies, based on the limitations addressed above, a recommendation can be made for supplementary studies that have a longer duration, are double‐blind and randomized in design, have a larger sample size with an equal distribution of both ethnicities and genders, and only include patients with schizophrenia. In addition, this review was conducted by one individual: to reduce author bias, more than one author should be used. Furthermore, it would also be useful for the controls, such as diet and exercise, to remain the same, preventing the impact of confounding variables. In this particular study, there was a variation of metformin doses used; thus, research is also required into the sufficient dose of metformin to be used. Further limiting the effect of confounding variables, further studies should utilize a single dose to be able to ascertain metformin's effects.

## 6 Conclusions and Summary

This comprehensive study was able to improve the database surrounding adjuvant metformin in reducing olanzapine‐induced metabolic adverse effects. Performing as a structured analysis, this review analyzed and narratively synthesized results from two main study types, open‐label and RCTs, across six studies. The lack of further evidence types can be explained by an absence of evidence related to the research question. Rationally, it looked at variables associated with metabolic syndrome including body weight/BMI, waist circumference, glucose/insulin level changes, and lipid profile.

Supporting current evidence, key findings display the robust beneficial effects of adjunctive metformin to reduce waist gain/BMI in patients taking olanzapine. Furthermore, although there was a lack of consistency among some variables, this study revealed metformin's ability to reduce triglycerides, LFC, and increase HDL. Coupled with the findings related to insulin resistance, this suggests a strategy to manage metabolic adverse effects and reduce the risk of progression to metabolic syndrome in patients taking olanzapine, particularly those with an elevated cardiovascular risk. Thus, in line with the proposed aims and objectives of this study, the beneficial evidence of this review supports the use of adjuvant metformin in reducing olanzapine‐induced metabolic adverse effects, fulfilling the aims related to assessing if adjuvant metformin is effective in reducing the metabolic adverse effects of olanzapine therapy. However, for further understanding, additional areas of research can be suggested, including determining an age, gender, and dose‐dependent effect.

In conclusion, this review supports the use of metformin as an adjunct therapy to olanzapine to reduce some of the metabolic adverse effects such as weight gain, reduction in triglycerides, LFC, and insulin resistance. Utilizing this combination in a clinical setting can be useful to lessen the metabolic adverse effects associated with olanzapine in patients diagnosed with schizophrenia.

## Author Contributions

Aquib Butt read all articles and formulated the PRISMA flow chart, analyzed relevant articles, wrote the whole article and checked references. Soban Sadiq designed project, wrote title, reviewed all articles, reviewed the analysis and whole manuscript.

## Ethics Statement

The authors have nothing to report.

## Consent

The authors have nothing to report.

## Conflicts of Interest

The authors declare no conflicts of interest.

## Supporting information


**Data S1:** npr270061‐sup‐0001‐Supinfo.docx.

## Data Availability

Data is available as supplementary file in public repository figshare DOI: https://doi.org/10.6084/m9.figshare.30081454.v1.
