# Supplementary material for: Does Adjuvant Metformin Reduce Olanzapine‐Induced Metabolic Adverse Effects in Patients Diagnosed With Schizophrenia
Source: Neuropsychopharmacol Rep. 2025 Sep 25;45(4):e70061. doi: 10.1002/npr2.70061 (PMC12463546; doi:10.1002/npr2.70061)
Supplement: Supplementary file 1 — Data S1: npr270061‐sup‐0001‐Supinfo.docx. [file NPR2-45-e70061-s001.docx]

**Title: Does adjuvant Metformin reduce Olanzapine-induced metabolic adverse effects in patients diagnosed with Schizophrenia**

Aquib Butt^1^ and Soban Sadiq^1^

^1^Kent and Medway Medical School, University of Kent, Canterbury, United Kingdom

Corresponding author: Aquib Butt ([a.butt2490@kmms.ac.uk](mailto:a.butt2490@kmms.ac.uk))

PICO tool

| Population | Adults diagnosed with Schizophrenia treated with Olanzapine |
| --- | --- |
| Intervention | Metformin |
| Comparison | No metformin |
| Outcome | Reduction in any metabolic adverse effects/parameters |
| My research question | Does adjuvant Metformin reduce Olanzapine-induced metabolic adverse effects in patients diagnosed with Schizophrenia |
| Potential IRP Hypothesis | *H_0_* Metformin does not reduce Olanzapine-induced metabolic adverse effects.  *H_1_* Metformin does reduce Olanzapine-induced metabolic adverse effects. |

## Search strategy

| Description of search | Details |
| --- | --- |
| Key words used, based on PICO analysis | #1 Metformin  #2 Adjunct  #3 Olanzapine  #4 Metabolic effects  #5 Schizophrenia |
| Boolean operator used “AND” | Long string format:  Metformin AND adjunct AND Olanzapine AND Metabolic effects AND Schizophrenia  Line format:  #1 Metformin  #2 Adjunct  #3 Olanzapine  #4 Metabolic effects  #5 Schizophrenia  #6 #1 AND #2 AND #3 AND #4 #5 |
| Synonyms and alternative words | Metformin:  Glucophage  Riomet  Glumetza  Adjunct:  Add-on  Addition  Combination  Olanzapine:  Zyprexa  Zalasta  ZypAdhera  Metabolic effects:  Waist circumference  Diabates  Lipids  Insulin  Triglycerides  Weight  LDL  Obesity  Schizophrenia |
| Boolean operator “OR” | Long string format:  (Metformin OR Glucophage OR Riomet OR Glumetza) AND (Adjunct OR add-on OR addition OR combination) AND (Olanzapine OR Zyprexa OR Zalasta OR ZypAdhera) AND (Metabolic effects OR Waist circumference OR Diabates OR Lipids OR Insulin OR Triglycerides OR Weight OR LDL OR Obesity) AND Schizophrenia  Line format:  #1 Metformin OR Glucophage OR Riomet OR Glumetza  #2 Adjunct OR add-on OR addition OR combination  #3 Olanzapine OR Zyprexa OR Zalasta OR ZypAdhera  #4 Metabolic effects OR Waist circumference OR Diabates OR Lipids OR Insulin OR Triglycerides OR Weight OR LDL OR Obesity #5 Schizophrenia  #6 #1 AND #2 AND #3 AND #4 #5 |
| Word variations and truncations* | Long string format:  (Metformin OR Glucophage OR Riomet OR Glumetza) AND (Adjunct OR add* OR combin*) AND (Olanzapine OR Zyprexa OR Zalasta OR ZypAdhera) AND (Metab* OR Waist circumference OR Diab* OR Lipid* OR Insulin OR Triglyceride* OR Weight OR LDL OR Obesity) AND Schizophrenia  Line format:  #1 Metformin OR Glucophage OR Riomet OR Glumetza  #2 Adjunct OR add* OR combin*  #3 Olanzapine OR Zyprexa OR Zalasta OR ZypAdhera  #4 Metab* OR Waist circumference OR Diab* OR Lipid* OR Insulin OR Triglyceride* OR Weight OR LDL OR Obesity  #5 Schizophrenia  #6 #1 AND #2 AND #3 AND #4 #6 |
| **Number of Hits:**  Embase =32  Psychinfo =5  PubMed =19  Scopus =15 | |
| Inclusion and exclusion criteria | Inclusion Criteria:   - Adults diagnosed with Schizophrenia and taking Olanzapine - Primary outcomes measures include metabolic parameters - Studies investigating adjunct Metformin on Olanzapine therapy - Quantitative data - Studies produced in the last 20 years - Clinical trials       Exclusion Criteria:   - Studies that use adjuncts other than Metformin - Studies that study Metformin with antipsychotics other than Olanzapine - Studies investigating more than one adjunct on the same subject - Animal studies - Studies with no data on metabolic outcomes |

## Final 6 articles chosen for the review

|  | Articles |
| --- | --- |
| 1. | Wu, R.-R., Zhao, J.-P., Guo, X.-F., He, Y.-Q., Fang, M.-S., Guo, W.-B., Chen, J.-D. and Li, L.-H. (2008) ‘Metformin Addition Attenuates Olanzapine-Induced Weight Gain in Drug-Naive First-Episode Schizophrenia Patients: A Double-Blind, Placebo-Controlled Study’, *American Journal of Psychiatry*, 165(3), pp. 352–358. Available at: <https://doi.org/10.1176/appi.ajp.2007.07010079>. |
| 2. | Rado, J. and von Ammon Cavanaugh, S. (2016) ‘A Naturalistic Randomized Placebo-Controlled Trial of Extended-Release Metformin to Prevent Weight Gain Associated With Olanzapine in a US Community-Dwelling Population’, *Journal of Clinical Psychopharmacology*, 36(2), pp. 163–168. Available at: <https://doi.org/10.1097/JCP.0000000000000469>. |
| **3.** | Baptista, T., Rangel, N., Fernández, V., Carrizo, E., El Fakih, Y., Uzcátegui, E., Galeazzi, T., Gutiérrez, M.A., Servigna, M., Dávila, A., Uzcátegui, M., Serrano, A., Connell, L., Beaulieu, S. and de Baptista, E.A. (2007) ‘Metformin as an adjunctive treatment to control body weight and metabolic dysfunction during olanzapine administration: A multicentric, double-blind, placebo-controlled trial’, *Schizophrenia Research*, 93(1–3), pp. 99–108. Available at: <https://doi.org/10.1016/j.schres.2007.03.029>. |
| 4. | Chen, C.-H., Chiu, C.-C., Huang, M.-C., Wu, T.-H., Liu, H.-C. and Lu, M.-L. (2008) ‘Metformin for metabolic dysregulation in schizophrenic patients treated with olanzapine’, *Progress in Neuro-Psychopharmacology and Biological Psychiatry*, 32(4), pp. 925–931. Available at: <https://doi.org/10.1016/j.pnpbp.2007.11.013>. |
| 5. | Baptista, T., Martínez, J., Lacruz, A., Rangel, N., Beaulieu, S., Serrano, A., Arapé, Y., Martinez, M., de Mendoza, S., Teneud, L. and Hernández, L. (2006) ‘Metformin for Prevention of Weight Gain and Insulin Resistance with Olanzapine: A Double-Blind Placebo-Controlled Trial’, *The Canadian Journal of Psychiatry*, 51(3), pp. 192–196. Available at: <https://doi.org/10.1177/070674370605100310>. |
| 6. | Wang, L., Chen, Y., Sui, Y.-C., Tan, X.-Q., Zhou, Z., Li, N. and Xu, L.-P. (2020) ‘Metformin Attenuates Liver Fat Content: Finding from Schizophrenia Patients with Olanzapine-induced Weight Gain.’, *Clinical psychopharmacology and neuroscience : the official scientific journal of the Korean College of Neuropsychopharmacology*, 18(1), pp. 67–74. Available at: <https://doi.org/10.9758/cpn.2020.18.1.67>. |

## Key characteristics of the studies in the review

| **Study** | **Type of study** | **Objective of study** | **Number of participants** | **Inclusion criteria** | **Length of study** | **Metabolic Outcome measured** | **Dose of olanzapine + Metformin assessed** | **Patient demographic** | **Statistical analysis performed** | **Limitations** |
| --- | --- | --- | --- | --- | --- | --- | --- | --- | --- | --- |
| (Wu et al., 2008) | Randomised, double blind placebo-controlled trial | Assess efficacy of Metformin for prevention of Olanzapine induced weight gain | 40 patients initially recruited but 37 (92.5%) completed treatment | 18-50  Drug naïve, first-episode schizophrenia diagnosed using the DSM-IV criteria  No antipsychotics, recreational drugs in the past 3 months before enrolment | 12 weeks | Body weight  Proportion of patients who gained more than 7% of their baseline weight at the end of 12 weeks  Waist-hip ratio  Fasting glucose and insulin  Insulin resistance index | Olanzapine:15mg  Metformin:  750mg/day  P | Olanzapine + Metformin:  Mean age: 25.4 +/- 3.9  Mean Duration of disease: 6.8 years +/- 3.1  Olanzapine + Placebo:  Mean age: 24.8 +/- 3.5  Duration of disease 7.6 years +/- 3.6 | Descriptive statistics including mean and SD. Categorical variables were described using frequencies and %. Students t test and chi squared analysis. ANCOVA. Intent to treat analysis  Pearsons’s correlation | Short duration  No long term follow up  Did not address whether behavioural and nutritional intervention with metformin could control body weight more effectively  Restricted diet to patients, which doesn’t reflect real-world  No examination of Olanzapine-Metformin pharmacokinetic interactions |
| (Rado and von Ammon Cavanaugh, 2016) | Randomized, double blind placebo-controlled pilot study | Assess efficacy, tolerability and safety of adjunctive metformin vs placebo for the prevention of Olanzapine-associated weight gain | 25 patients | 18+  Diagnosed with schizophrenia, schizoaffective disorder, bipolar or major depression. | 24 weeks | Weight measures (baseline to end point changes in weight and BMI and percent weight change within and between groups)  Insulin resistance measured by baseline to end point changes in the homeostatic model assessment for insulin resistance (HOMA-IR)  Secondary outcomes: waist circumference, glucose parameters (FBG, OGTT, HbA1C) and fasting lipid | Mean dose of olanzapine used was 16mg  Metformin extended release titrated to 2000mg as tolerated over 24 weeks | Olanzapine +Metformin:  7 males  5 females  Race: White 7  African American 4  Other 1  Mean age: 33.5 +/- 10.12  Months ill 9.33 +/- 6.69  Olanzapine +Placebo:  5 males  8 females  Race:  3 white  1 African American  2 other  Mean age 39.08 +/-8.62  Months ill:11.58 +/- 8.37 | Intent to treat analysis  Independent t-tests  Chisquare tests | Small sample size  Naturalistic design introduces more confounding variables such as patients in the Olanzapine + Metformin group were on concomitant psychotropic medications at baselines and more psychotropic medications were added. This would favour more weight gain in the Olanzapine + Metformin group compared with the Olanzapine + placebo group |
| (Baptista et al., 2007) | Double blind randomized controlled trial | To assess whether Metformin prevents or reverses body weight gain in patients with schizophrenia or bipolar disorder under Olanzapine administration | 80 patients initially enrolled  72 completed the study | Older than 18  Free from any hormonal replacement therapy and chronic diseases besides the mental disorder and normal laboratory results including (liver, kidney, thyroid and fasting glucose)  Olanzapine monotherapy for 4 months | 12 weeks | Anthropometric variables: Weight loss, body weight, body mass index and waist circumference.  Biochemical variables: glucose, insulin, leptin, cortisol, growth hormone, fibrinogen, c-reactive protein, HbA1c and index of insulin resistance (HOMA-IR) | Mean dose of Olanzapine: 10.3mg +/- 2.2  No Mean stated but patients randomly allocated to 850mg-2250mg | Metformin group:  36 participants  23 male average age of 42.4+/- 11.7  13 female average age 46.2 +/- 11.3  Placebo group:  19 male average age 43.2 +/- 14.5  17 females average age 46.0 +/-9.1 | Data analysis estimated with the one-sample Kolmogorov-Smirnov test and Levene’s test for quality of variances.  Within and between group comparison was done using the two tailed t-test.  Two-side test were used to minimize the alpha error/ Bivariate correlation analysis was conducted using the Pearson and Spearman coefficients  Frequencies were analysed using the chi-squared test. With results considered significant at the p<0.05 level | Short follow up period, only 12 weeks.  Heterogeneity of basal BMI and previous Body Mass Gain in patients in the study |
| (Chen et al., 2008) | Open label, prospective multi-centre study | Assess the reversal effects of Metformin on metabolic disturbance induced by Olanzapine among Schizophrenic patients | 24 | 16-60 in patients  Fasting glucose of 126mg/dL or less and no personal or family history of diabetes. Diagnosed with Schizophrenia. Drugs that may influence bodyweight, glucose/lipid metabolism or drug disposition (lithium, carbamazepine , valproic acid, propranolol, triclyclic antidepressant or SSRI ) were not allowed. Had have received Olanzapine for 3 months | 8 weeks | Body weight  BMI  Fasting glucose levels  Fasting insulin levels  Triglycerides  Total cholesterol  HDL  LDL  Leptin levels  Insulin secretion (using an intravenous glucose tolerance test )  HOMA-IR  HOMA-B  Glucose disappearance rate (kG)  Metabolic syndrome based on modified ATP III criteria for Asians | Mean dose of Olanzapine: was 11.5+/- 3mg/day  Metformin: 1500mg/day | Mean age of sample 40.3+/-9.7 years  All subjects were Taiwanese and 16 were men 8 female | Kolmogorov-Smirnov testing to assess distribution of the variables. Subgroups compared using t-test for continuous variables and chi-square test for categorical variables.  Base line Analysis of covariance (ANCOVA) for all outcomes including body weight, BMI and laboratory assessments  P<0.05 was considered statistically significant | Open label so participants and researchers knew when metformin was being used, which could introduce a placebo effect.  No placebo-controlled group; unable to exclude that recruited patients had strong motivation to reduce body weight and change their lifestyle in the study.  Short duration of Metformin exposure- limited to 8 weeks.  Small sample size |
| (Baptista et al., 2006) | Double blind, randomized placebo-controlled trial | To assess if Metformin prevents body weight gain and metabolic dysfunction in patients with Schizophrenia treated with Olanzapine | 40 | Clinically stable inpatients  With severe schizophrenia or schizoaffective disorder free from any other chronic disease and HRT.  Required to have normal physical and laboratory examination results including ( liver, renal, haematology and thyroid function | 14 weeks | Body weight  BMI  Waist circumference  Glucose  Brief Psychiatric Rating scale  Lipids  Post load glucose levels  HOMA-IR | Olanzapine 10mg  Metformin 850-1750mg | 22 men (mean age 47.9 +/- 10.6) and 15 women (mean age 47.4 +/- 5.9)  Metformin group 9 women and 10 men  Placebo group 6 women and 12 men | Two tailed t tests for unpaired and paired samples were conducted for between and within group comparison respectively.  Bivariate correlation calculated between BPRS scores, anthropometric variables and chemical variables  When P<0.05 values were considered significant | Low dose of Olanzapine, small population size and short duration of study. Sample of patients may not represent most patients with schizophrenia taking atypical antipsychotic drugs |
| (Wang et al., 2020) | Randomized, double blind placebo-controlled trial | To evaluate the efficacy of Metformin on Liver fat content, in first episode schizophrenia patients with Olanzapine induced weight gain | 36 entered 5 withdrew within 4 weeks  Leaving 31 | Under Olanzapine monotherapy with a stable dose for at least 2 months  Had been maintained on the same daily doses of Olanzapine for at least 4 week prior to study enrolment  We’re willing to lose weight or prevent excessive body weight gain  Had to have a relatively stable improvement , daily alcohol consumption <20g/day (men) or 10g/day (women)  Participants gained more than 7% of their pre-drug body weight.  First episode schizophrenia | 16 weeks | Anthropometric measures including weight, height, BMI, Waist circumference and waist-height ratio (WHR).  Liver Fat content measured using MRI  Glucose and Lipid metabolism homeostasis model assessment of Insulin resistance index (HOMA IR), were measured analysing the pattern between the change value of LFC and other indicators | Dose of Metformin was 1000mg/day  For the first 7 days 250mg TDS  Then 500mg BD  Does not specify the single dose of Olanzapine used across all participants. But in the inclusion criteria it states they included participant if they were on a stable dose of Olanzapine between 5mg/day and 30mg/day | Average age of patients in the Metformin group 28.44 +/- 8.92  Average age of patients in the placebo group 29.27+/- 7.44  No genders listed | Baseline characteristic of metformin and placebo group were compared using t test for independent samples.  Students t test to evaluate between group differences in changes of LFC from base line to end point and changes of body weight BMI, and WHR.  Main analysis of covariance (ANCOVA) with repeated measurements.  Relationship between LFC and metabolic variables were assessed with Pearson and Spearman correlations.  A p value <0.05 was considered statistically significant. | Small sample size  Potential gender bias  Short duration of study  LFC measurement; MRI is expensive to do and technically demanding thus not suitable for clinical practise. |

## Metabolic outcomes and reported adverse drug reactions of each study analysed in the review

|  | Body weight/BMI | Waist Circumference | Glucose/insulin level changes | Lipid profile | Other metabolic outcomes measured | ADR |
| --- | --- | --- | --- | --- | --- | --- |
| (Wu et al., 2008) | **Statistically Significant findings:**   - **Increase in weight and BMI in Olanzapine + placebo group were significantly greater compared to Olanzapine + Metformin at the end of the 12 weeks**   From baseline to 12 weeks O/P group had a mean increase in weight of 6.87kg ± 4.23 whereas the O/M group had a mean increase of 1.9kg±2.72. Statistically significant between group difference of *p*<.02  From baseline to 12 weeks the O/P group had a mean increase in BMI of 2.26 ±1.12 compared with the O/M group whereas the O/M group had a mean increase in BMI of 0.54 ±0.92. Statistically significant between group difference of *p*<.01   - Fewer patients in the Metformin group (N=3 16.7%) compared with placebo group (N=12 63.16%) increased their initial body weight by 7% ( cut off for clinically significant weight gain) which was a significant difference (*p*<.001) - Significant group effect in ANCOVA weight and BMI; weight: F=9.87, df=1, 35, *p*<.01; body mass index: F=8.89, df=1, 35, *p*<.01   Insignificant findings:   - No significant changes found in elevated BMI between both groups | **Statistically significant findings:**   - **Increase in waist circumference and reached statistical significance within the two groups in week 2, 4, 8 and 12**   O/M group in weeks 2, 4, 8 and 12 had waist circumference increases of (cm): 0.11±0.05, 0.22±0.1, 0.32±0.12 and 0.46±0.14 respectively (*p*<.05)  O/P group in weeks 2,4,8 and 12 had waist circumference increases of (cm):0.15±0.08, 0.48±0.15, 0.78±0.26 and 1.37±0.62 respectively (*p*<.05)  Insignificant findings:   - Increase in waist circumference and waist to hip ratio was not significantly different between both groups | **Statistically significant findings:**   - **Mean fasting insulin, and insulin resistance index increasing significantly in Olanzapine plus placebo group in weeks 8 and 12**   From baseline fasting insulin at 8 and 12 weeks increased 4.82µIU/ml±2.97 and 6.78µIU/ml±3.29 (*p*<.05)  From baseline insulin resistance index at 8 and 12 weeks increased 1.03 ±0.63 and 1.49±0.67 respectively (*p*<.05)   - **At week 8 and 12 increases in fasting insulin and insulin resistance index of Olanzapine plus placebo were significantly greater compared to Olanzapine plus Metformin**   With O/M group having an increase in fasting insulin of 1.23±2.82 µIU/ml and 0.81±2.95 µIU/ml at weeks 8 and 12 respectively and an increase in insulin resistance index of 0.29±0.62 and 0.22±0.66 respectively. A between group comparison of O/M and O/P group of increase in insulin revealed a t of -7.352 and *p*<.01 and t of -9.075 and *p*<.01 for weeks 8 and 12 respectively. For insulin resistance index comparison between both groups O/M group at 8 and 12 weeks, week 8 had a t score of -7.724 and *p*<.01 and -10.081 and *p*<.01 for week 12   - **Significant group effect found in ANCOVA (insulin: F=64.27, df=1, 35, *p*<.01; insulin resistance index: F=98.21, df=1, 35, *p*<.01)**   Insignificant findings:   - No significant changes to insulin and insulin resistance index were detected in the Olanzapine + placebo group | NA | NA | Not listed |
| (Rado and von Ammon Cavanaugh, 2016) | **Statistically significant changes:**   - **Weight change (*p*<.05), change in BMI (*p*<.045) and % change in weight *(p*<.037) from baseline were statistically greater in the Olanzapine and placebo group compared to the Olanzapine and Metformin group**   The O/M group had a mean increase in weight of 2.54±2.35 kg whereas the O/P group had a mean increase of 5.88±5.23kg a statistically significant comparison of *p*<.05  The O/M had a mean increase in BMI of 0.85±0.76 and the O/P had a mean increase of 2.02±1.77 , a statistically significant comparison of *p*<.045 | **Insignificant changes:**   - **Greater increase in waist circumference in O/P group compared to O/M group (*p*<.085)**   The O/M group had a mean increase of 0.35±2.57 whereas the O/P group had a mean increase of 2.56±3.44 which was a of *p*<.085 | **Statistically significant changes:**   - **Greater increase in HOMA-IR in the O/P group compared with O/M (*p*<.097)**   The O/M group had a change of -0.73±1.65 whereas the O/P group had a change of 1.13±2.76 which had a statistical significance of *p*<.097  Insignificant changes:   - FBG, OGTT and HbA1c   Mean FBG change for O/M was 7±16.64 and 8.54±27.29 for O/P *p*<.868  Mean OGTT change for O/M was 6.78±24.30 and for O/P was 14.33±25.01 *p*<0.525  Mean HbA1c change was -0.5±0.28 for O/M group compared with 0.1±0.26 for the O/P group *p*<.262 | **Insignificant changes**   - No statistically significant changes found in Cholesterol, LDL, HDL and Triglycerides   O/M group had a mean cholesterol change of 10.5±27.92 compared with 8.80±12.55 in the O/P group a *p* value of <.262  O/M group had a mean LDL change of 13±21.62 compared with 1.33 in the O/P group *p*<.863  O/M group had a mean HDL change of 4.0±10.02 compared with 2.36±4.8 in the O/P group *p*<0.647 |  | GI upset  1 drop out for drowsiness and another for insomnia in the O/M group |
| (Baptista et al., 2007) | **Statistically significant changes**:   - **The metformin group statistically lost more body weight compared to the placebo group**   The Metformin group had a mean change in weight of -1.4±3.2kg (*p*=.01) whereas the placebo group had a mean of -0.18±2.8kg(*p*=.7)  Between group analysis was marginally significant (*p*=.09)   - BMI had a statistically significant decrease in the Metformin group but not after placebo – between group comparison was not significant   The metformin group had a mean change in BMI of -0.47±1.2 *p*=.01 compared with the placebo group who had a mean change of -0.07±1.1 *p*=.6. A between group analysis was not statistically significant (*p*=.1)  Insignificant findings   - Between group comparison of BMI and body weight | Insignificant changes:   - No statistically significant differences were found in waist circumference   Metformin group had a mean change in waist circumference of -0.1±5.9cm *p*=0.9.  Placebo group had a mean change in waist circumference of 0.5±5.6cm *p*=0.6 | **Statistically significant changes:**   - **Insulin and HOMA-IR significantly increased after placebo (p=0.001 and 0.006 respectively)**   O/M group had a mean change of Insulin of 0.04±15.3 µIU/ml *p*=0.9 compared with 4.9±7.2 µIU/ml *p*=0.001  O/M group had a mean change of HOMA-IR of -0.09±3.1 *p*=0.8 compared with a change of 0.91±1.7 in the placebo group *p*=0.006   - Increase in HbA1c in the Metformin group (*p*=0.011) but not placebo group   O/M group had a mean change of 0.47±1.1 *p*=0.01 compared with the placebo group who had a change of 0.42±1.5 *p*=0.11  Insignificant changes:   - Between group comparison of Glucose(*p*=0.5), insulin (*p*=0.1), and HOMA-IR(*p*=0.1) - Between group comparison of weight change(*p*=0.09) and BMI(*p*=0.1). | **Statistically significant changes:**   - **HDL decreased in the Metformin group (within group comparison *p*=.007)**   The O/M group had a mean HDL change of -5.8±12.0mg/dL *p*=.007 compared with the placebo group whom had a change of -2.0±13.9mg/dL *p*=.4  Insignificant changes:   - No statistically significant changes were observed in cholesterol and triglyceride levels | **Statistically Significant changes**   - **Leptin decreased after metformin (*p*=0.09) but remained stable after placebo- intergroup analysis reached marginal significance (*p*=0.07)** - **Cortisol levels increased in both groups (*p*=0.051 and 0.1 for metformin and placebo respectively)**   Insignificant changes:   - Between group analysis of cortisol (*p*=0.3) - Growth hormone changes, (between group analysis *p*=0.4) | GI upset |
| (Chen et al., 2008) | **Statistically significant findings:**   - **The decrease in mean body weight and BMI reached statistical significant after 8 weeks of metformin (p<.01)**   At 8 weeks of metformin therapy the mean change in body weight was -2.2±1.8kg which was statistically significant (p<.01) | NA | **Statistically significant findings:**   - **Fasting glucose significantly decreased after 8 weeks of Metformin(p<0.01)**   After 8 weeks the mean change in fasting glucose was -4±1.5mg/dL   - **Fasting insulin level significantly decreased at week 8 (p<0.01)**   Mean decrease of 3.7±14.53   - **Insulin secretion(-1323±2871.69 p<0.01), beta cell function (HOMA-B index;153.6± 239.1 p>0.01) and insulin resistance (HOMA-IR index1.57±2.92 p<0.01) significantly decreased after 8 weeks of Metformin**   Insignificant findings:   - Glucose disappearance rate   At baseline there was a 1.56±0.31 mmol/l per minute compared with 1.5±0.33mmol/L per minute. | **Statistically significant findings:**   - **Triglycerides significantly decreased at 8 weeks after Metformin trial (*p*<.01)**   A comparison from baseline to 8 weeks showed a change of -38.1±104.3 mg/dL  Insignificant changes:   - No significant changes in the levels of total cholesterol, HDL, LDL and Leptin were observed in the treatment period | No statistical difference in the prevalence of metabolic syndrome or individual metabolic syndrome was found | Nausea |
| (Baptista et al., 2006) | Insignificant findings:   - Body weight increased similarly in the metformin and placebo group   From baseline the mean change in weight in the Metformin group was 5.5kg±3.3  Compared with 6.3±2.3 in the placebo group  **Statistically significant findings:**   - **Within group analysis (paired t test) was statistically significant for weeks 7 and 14. For the metformin group t_18_=5.9, *p*<.001 and t_18_=7.3,*p*<.001. For the placebo group t_17_=6.2, *p*=.001 and t_17_=6.3, *p*=.001.** - **BMI increased significantly in both groups (*p*<.01) but no between group differences ( data not shown)** | **Statistically significant findings:**   - **Waist circumference increased significantly in both groups but there was no between group difference** | **Statistically significant findings:**   - Basal glucose levels decreased under metformin (p<0.02) but remained stable after taking placebo   At baseline the mean basal glucose was 4.8±0.5 and at week 14 it reduced to 4.4±0.6mmol/L compared with the placebo group whom had a mean baseline glucose of 4.7±0.3mmol/L and a week 14 mean of 4.6±1.0   - **Insulin decreased significantly in both groups reflected by the HOMA-IR (Metformin group p<0.001 and placebo group p<0.01**)   At baseline the Metformin group had a mean HOMA-IR of 4.5±1.3 and a week 14 of 2.9±1.3. Compared with the placebo group whom had a baseline mean HOMA-IR of 4.9±1.7 and at week 14 3.1±1.7  Insignificant findings:   - No significant changes observed in post load glucose levels | **Statistically significant findings:**   - Triglycerides increased significantly after taking Metformin (*p*<0.001) but not after placebo   At baseline the mean triglycerides was 112±56.6 and at week 14 this value increased to 123±64.7 mg/dl. Compared with the placebo group who had a baseline mean of 142±87.7 and a week 14 of 153±86.8 mg/dl   - **Total cholesterol decreased after taking Metformin *p*<.001) but not after placebo (*p*=.02)**   In the Metformin group the mean baseline cholesterol was 199±33.2 mg/dl at week 14 the mean was 181±38.6. Compared with the placebo group; mean baseline of 194±38.7 and a week 14 of 205±66.2 mg/dl   - **LDL cholesterol significantly increased after taking placebo (*p*<.05)**   The metformin group had a mean baseline of 130±33.4 and at week 14 102±40.3 compared with the placebo group; baseline of 116±30.7 and week 14 of 128±65.7   - HDL cholesterol increased in both groups (metformin *p*<.02, and placebo p=.001)   The Metformin group had a mean baseline of 50.1±9.8 and at week 14 the mean was 54.5±9.4  Compared with the placebo group where the baseline was 45.3±10.3 and week 14 48.3±10.1  Insignificant findings:   - LDL cholesterol decreased after taking Metformin but not significanty (*p*=0.1) - VLDL levels did not significantly change in either group. |  | Gastrointestinal discomfort |
| (Wang et al., 2020) | NA | NA | **Statistically significant findings:**   - H**OMA-IR decreased after intervention (t=2.166, p=0.047)**   **Before intervention the mean HOMA-IR was 3.5±0.6 and after intervention it was 3.1±0.4**  Statistically insignificant findings:   - HbA1c decreased after intervention (t 0.309. p=0.762)   Before intervention the mean HbA1c was 5.0±0.6 and after intervention it was 4.9±0.5   - FPG increased after intervention (t=0.616, p=0.543)   Before intervention 5.3±0.2 mmol/L and after intervention it was 5.5±0.2 mmol/L | **Statistically significant findings:**   - Triglyceride levels decreased after Metformin interventiont=2.242, *p*=.041   Before intervention the mean was 2.4±0.7 and after intervention it was 2.1±0.6   - HDL cholesterol increased after metformin treatment (*p*=.046)   Before intervention the mean was 1.1±0.4 mmol/L and after intervention it was 1.6±0.5 mmol/L  Statistically insignificant findings:  Total cholesterol decreased after intervention(*p*=0.329)  LDL cholesterol decreased after intervention *p*=0.228 | **Statistically significant findings:**   - **Liver fat content change across the 16 weeks was -2.91% for the metformin group and 0.59 for the placebo group with a between-group difference of -3.5% (p=0.009)** - **The between group difference showed a significant time-by-treatment interaction (p=0.009**) | Nausea |

CASP systematic review checklist adapted from CASP [51]

|  | (Wu et al., 2008) | (Rado and von Ammon Cavanaugh, 2016) | (Baptista et al., 2007) | (Baptista et al., 2006) | (Wang et al., 2020) |
| --- | --- | --- | --- | --- | --- |
| Section A is the basic study design valid for a randomised controlled trial? | | | | | |
| 1.Did the study address a clearly formulated research question? | **Yes**/Cant tell/No  Assessed the efficacy of Metformin in preventing Olanzapine-induced weight gain in drug-naive first episode patients with Schizophrenia | **Yes**/Cant tell/No  To examine the use of Metformin to prevent Olanzapine-associated weight gain and metabolic disturbances over a 6month period in a US community | **Yes**/Cant tell/No  Assesses the effects of Metformin on bodyweight in adult patients under prolonged Olanzapine administration | **Yes**/Cant tell/No  To assess wheather metformin prevents body weight gain in patients with schizophrenia who are treated with Olanzapine | **Yes**/Cant tell/No  To evaluate the efficacy of Metformin on liverfat content in first episode schizophrenia patients with Olanzapine induced weight gain |
| 2. Was the assignment of participants to interventions randomised? | **Yes**/Cant tell/No  Using a computer generated table to one of the two treatments | **Yes**/Cant tell/No  Using a computer based algorithm to 1 of 2 treatments (Olanzapine plus metformin or Olanzapine plus placebo) | **Yes**/Cant tell/No  Subjects were randomly assigned via a computer based program to metformin or identical placebo pill. | **Yes**/Cant tell/No  Using computer based random allocation of patients to either Olanzapine plus Metformin or Olanzapine plus placebo . | **Yes**/Cant tell/No  Particiapants were randomly assigned to treatment condition based on a computer generated sequence of numbers |
| 3. Were all participants who entered the study accounted for at its conclusion? | Yes/Cant tell/No  40 entered the trial and 3 withdrew within the first 4 weeks due to a lack of response( 1 placebo patient, 2 metformin patients) | **Yes**/Cant tell/No  1 drop out for drowsiness, another for insomnia | **Yes**/Cant tell/No  7 subjects abandoned the study due to changing residence, and one relapsed | **Yes**/Cant tell/No  2 patients taking placebo and one taking Metformin dropped out due to change in residence | **Yes**/Cant tell/No  5 withdreew within the first 4 weeks due to an exacerbation of psychosis |
| Section B Was the study methodologically sound? | | | | | |
| 4. (a) Were the participants ‘blind’ to intervention they were given? | **Yes**/Cant tell/No  Medication provided in coded containers containing the identical appearing pills | **Yes**/Can’t tell/No  Metformin and placebo were identical in appearance and were given to patients in coded containers by a separate research pharmacy | **Yes**/Can’t tell/No | Yes/**Can’t tell**/No | **Yes**/Cant tell/No  Allocation was concealed using sealed opaque envelopes |
| (b) Were the investigators ‘blind’ to the intervention they were giving to participants? | **Yes**/Cant tell/No  Concealment was carried out by a research pharmacist in a separate facility | **Yes**/Cant tell/No  Tablets were provided my a separate research pharmacy | **Yes**/Can’t tell**/No**  Alhtough in the outpatient group, any dose adjustment was done by the study coordinator who was the only team’s member to know the individual treatment. | **Yes**/Can’t tell/No  The trial was a double-blind trial, but no further information was provided as to how allocation was concealed from investigators | **Yes**/Cant tell/No  The trial was a double-blind trial, but no further information was provided as to how allocation was concealed from investigators |
| (c) Were the people assessing/analysing outcome/s ‘blinded’? | Yes/**Cant tell**/No | Yes/**Can’t tell**/No | Yes/**Cant tell**/No | Yes/**Can’t tell/**No | Yes/**Cant tell**/No |
| 5. Were the study groups similar at the start of the randomised controlled trial? | **Yes**/Cant tell/No  The two treatment groups did not different significantly in demographic or clinical characteristics | Yes/Can’t tell/**No**  There were more females than males and more African americans than anyother race in the placebo group.  Mean baselilne for BMI was 29(4.4) for females versus 31.2(8,2) for males where as the mean baseline BMI for African Americans was 30.9 (.3) vs 29.1(4.2) for other races | **Ye**s/Can’t tell**/No**  No differences in previous body weight gain (P=0.49) Olanzapine dose (p=0.11) and treatment duration before the study (p=0.47) were observed in the subjects assigned to the Metformin and placebo groups.  Gender and age did not differ between the subjects who completed the study. However nothing was said regarding the patients ethnicities, and 76 patients had Schizophrenia and 4 had Bipolar | **Yes**/Can’t tell/No  Neither group differed significantly in other at baseline | **Yes**/Can’t tell/No  Demographic data collected showed that there was no difference interms of age, height, weight, waist circumference, and waist-to-hip ratio |
| 6. Apart from the experimental intervention, did each study group receive the same level of care (that is, were they treated equally)? | **Yes**/Can’t tell/No  There was nothing in the study methods to suggest the treatment group was treated differently to the placebo group and vice versa, | **Yes**/Can’t tell/No  There was nothing in the study methods to suggest the treatment group was treated differently to the placebo group and vice versa, | Yes/Can’t tell/**No**  Outpatients were given a 2-week treatment supply and had a weekly phone contact with a specific team researcher to control for side effects. | Yes/**Can’t tell/**No  The study does not discuss how the individual groups were treated | **Yes/**Can’t tell/No  There was nothing in the study methods to suggest the treatment group was treated differently to the placebo group and vice versa, |
| Section C: What are the results? | | | | | |
| 7. Were the effects of intervention reported comprehensively? | **Yes**/Cant tell/No  Primary outcomes included the changes in weight, BMI, waist-to-hip ratio, fasting glucose, fasting insulin, proportion of patients who gained more than 7% of their baseline body weight at 3 months and insulin resistance index, calculated based on the formula of homeostasis assessment for insulin resistance model: fasting insulin (10^3^ mIU/liter) x fasting glucoe (mmol/liter)/22.5  Secondary outcomes included changes in the scale for the assessment of Negative Symptoms (SANA) and Scale for the Assessment of Negative Symptoms (SANS) | **Yes**/Cant tell/No  The study reported changed in baseline of primary outcomes including weight and BMI, insulin resistance as HOMA-IR. It also reported changes from baseline of secondary outcomes including waist circumference, glucose parameters (FBG, OGTT, HbA1c) and fasting lipids | **Yes**/Cant tell/No  The study reported the changes in body weight, waist circumference and BMI. As well as changes in glucose, insulin, HOMA-IR, HbA1c, Leptin, cortisol and growth hormone | **Yes**/Can’t tell/No  Brief Psychiatric Rating Scale (BPRS)  Body weight  BMI  Waist circumference  Was reported at baseline, week 7 and week 14. | **Yes**/Cant tell/No  The study reported in primary outcome variables including fasting plasma glucose, HbA1c, triglycerides, total cholesterol, HDL-C, LDL-C and HOMA-IR. It also reported changes from baseline in Liver fat content. It also reported a pearsons correlation analysis of changes in lilver fat content and glycolipid metabolism indexes before and after metformin |
| 8. Was the precision of the estimate of the intervention or treatment effect reported? | **Yes**/Cant tell/No  Differences were considered statistically significant at p<0.05 | **Yes**/Cant tell/No  Differences were considered statistically significant when p<0.05 | **Yes**/Can’t tell/No  Results were considered significant at the p<0.05 level | **Yes**/Can’t tell/No  When p<0.05 values were considered significant | **Yes/**Can’t tell/No  A p value of <0.05 was considered statistically significant |
| 9. Do the benefits of the experimental intervention outweigh the harms and costs? | Yes/**Cant tell**/No  Cost not reported  Side effects not reported | Yes/**Cant tell**/No  Cost of intervention not reported | Yes/**Can’t tell**/No  Cost not reported | Yes/**Can’t tell**/No  Cost not reported  Only side effects reported were mild gastrointestinal side effects | Yes/**Can’t tell**/No  **No mention of costs or harm in the study** |
| Section D: Will the results help locally? | | | | | |
| 10. Can the results be applied to your local population/in your context? | **Yes**/Can’t tell/No  Participants were recruited from a Mental Health institute in china, with no more information regarding their background. However the results are applicable for this study | Yes/**Can’t tell**/No  The study included patients with the following psychiatric conditions, schizophrenia, schizoaffective disorder and major depressive disorder with psychotic features. All taking Olanzapine. The study did not demarcate data solely on schizophrenia patients | Yes/**Can’t tell**/No  The study used inpatients and outpatients in Venezuela; however it included both 76 Schizophrenia, 4 patients with bipolar and no description of ethnicity was given | Yes/**Can’t tell**/No  Patients had switched from conventional to atypical antipsychotics, and were treated with APD for an average of 30.7 years | Yes/Can’t tell/**No**  The study was limited by a small sample size. |
| 11. Would the experimental intervention provide greater value to the people in your care than any of the existing interventions? | **Yes**/Can’t tell/No  The study demonstrated that it is possible to attenuate antipsychotic-induced weight gain in drug-naïve young people with metformin, this intervention was also safe and well tolerated . However no discussion of finances, skills development or training needs are mentioned. | Yes/**Can’t tell**/No  Although the study demonstrated that metformin’s effectiveness for the prevention of Olanzapine induced weight gain, it does not give data specifically for the patients with schizophrenia | Yes/**Can’t tell**/No  The study states that their results encourage additional studies in a more homogenous population | **Yes**/Can’t tell/No  Aside from the ability of Metformin to prevent Olanzapine induced weight gain and affect triglyceride levels, it showed to display positive metabolic effects. | Yes/**Can’t tell**/No  The study has provided preliminary evidence that metformin is effective in promoting reducing Liver fat content and weight induced by Olanzapine in patients with first episode schizophrenia but it is limited by a small sample size. Furthermore the study does not provide cost related data. |

Cochrane risk of Bias **domains 1-5** [52]

Y=YES

PY=Probably yes

PN=Probably no

N=no

NI=No information

**Domain 1: Risks of bias arising from the randomisation process**

| Signalling questions | (Wu et al., 2008) | (Rado and von Ammon Cavanaugh, 2016) | (Baptista et al., 2007) | (Baptista et al., 2006) | (Wang et al., 2020) | Response Questions |
| --- | --- | --- | --- | --- | --- | --- |
| 1.1 Was the allocation sequence random? | **Y** | **Y** | **Y** | **Y** | **Y** | Y / PY / PN / N / NI |
| 1.2 Was the allocation sequence concealed  until participants were enrolled and  assigned to interventions? | **Y** | **Y** | **Y** | **Y** | **Y** | Y / PY / PN / N / NI |
| 1.3 Did baseline differences between  intervention groups suggest a problem with  the randomization process? | **N** | **N** | **N** | **N** | **N** | Y / PY / PN / N / NI |
| Risk-of-bias judgement | **Low** | **Low** | **Low** | **Low** | **Low** | Low / High / Some concerns |
| **Optional: What is the predicted direction of**  **bias arising from the randomization process?** | **NA** | **NA** | **NA** |  | **NA** | **NA / Favours experimental /**  **Favours comparator / Towards**  **null /Away from null /**  **Unpredictable** |

**Domain 2: Risk of Bias due to deviations from the intended interventions (effect of assignment to intervention)**

| Signalling questions | (Wu et al., 2008) | (Rado and von Ammon Cavanaugh, 2016) | (Baptista et al., 2007) | (Baptista et al., 2006) | (Wang et al., 2020) | **Response Options** |
| --- | --- | --- | --- | --- | --- | --- |
| 2.1. Were participants aware of their  assigned intervention during the trial? | **N** | **N** | **N** | **N** | **N** | Y / PY / PN / N / NI |
| 2.2. Were carers and people delivering the  interventions aware of participants assigned intervention during the trial? | **N** | **N** | **N** | **N** | **N** | Y / PY / PN / N / NI |
| **2.3. If Y/PY/NI to 2.1 or 2.2:** Were there  deviations from the intended intervention  that arose because of the trial context? |  |  |  |  |  | NA / Y / PY / PN / N / NI |
| **2.4 If Y/PY to 2.3:** Were these deviations  likely to have affected the outcome? |  |  |  |  |  | NA / Y / PY / PN / N / NI |
| **2.5. If Y/PY/NI to 2.4:** Were these  deviations from intended intervention  balanced between groups? |  |  |  |  |  | NA / Y / PY / PN / N / NI |
| 2.6 Was an appropriate analysis used to  estimate the effect of assignment to  intervention? | **Y** | **Y** | **Y** | **Y** | **Y** | Y / PY / PN / N / NI |
| **2.7 If N/PN/NI to 2.6:** Was there potential  for a substantial impact (on the result) of  the failure to analyse participants in the  group to which they were randomized? |  |  |  |  |  | NA / Y / PY / PN / N / NI |
| **Risk-of-bias judgement**  **Optional: What is the predicted direction of**  **bias due to deviations from intended**  **interventions?** | **Low** | **Low** | **Low** | **Low** | **Low** | Low / High / Some concerns  NA / Favours experimental /  Favours comparator /  Towards null /Away from  null / Unpredictable |

**Domain 2: Risk of bias due to deviations from the intended interventions (effect of adhering to intervention)**

| **Signalling questions** | (Wu et al., 2008) | (Rado and von Ammon Cavanaugh, 2016) | (Baptista et al., 2007) | (Baptista et al., 2006) | (Wang et al., 2020) | Response options |
| --- | --- | --- | --- | --- | --- | --- |
| 2.1. Were participants aware of their  assigned intervention during the trial? | **N** | **N** | **N** | **N** | **N** | Y / PY / PN / N / NI |
| 2.2. Were carers and people delivering the  interventions aware of participants  assigned intervention during the trial? | **N** | **N** | **N** | **N** | **N** | Y / PY / PN / N / NI |
| 2.3. [If applicable:] If Y/PY/NI to 2.1 or 2.2:  Were important non-protocol interventions  balanced across intervention groups? |  |  |  |  |  | NA / Y / PY / PN / N / NI |
| 2.4. [If applicable:] Were there failures in  implementing the intervention that could  have affected the outcome? |  |  |  |  |  | NA / Y / PY / PN / N / NI |
| 2.5. [If applicable:] Was there non-  adherence to the assigned intervention  regimen that could have affected  participants’ outcomes? | **N** | **N** | **N** | **N** | **N** | NA / Y / PY / PN / N / NI |
| **2.6. If N/PN/NI to 2.3, or Y/PY/NI to 2.4 or**  **2.5:** Was an appropriate analysis used to  estimate the effect of adhering to the  intervention? |  |  |  |  |  | NA / Y / PY / PN / N / NI |
| **Risk-of-bias judgement** | **Low** | **Low** | **Low** | **Low** | **Low** | Low / High / Some concerns |
| Optional: What is the predicted direction of  bias due to deviations from intended  interventions? | **NA** | **NA** | **NA** | **NA** | **NA** | NA / Favours experimental /  Favours comparator /  Towards null /Away from  null / Unpredictable |

**Domain 3: Missing outcome data**

| Signalling questions | (Wu et al., 2008) | (Rado and von Ammon Cavanaugh, 2016) | (Baptista et al., 2007) | (Baptista et al., 2006) | (Wang et al., 2020) | Response options |
| --- | --- | --- | --- | --- | --- | --- |
| 3.1 Were data for this outcome available  for all, or nearly all, participants  randomized? | Y | Y | Y | Y | Y | Y / PY / PN / N / NI |
| 3.2 **If N/PN/NI to 3.1**: Is there evidence that  the result was not biased by missing  outcome data? |  |  |  |  |  | NA / Y / PY / PN / N |
| 3.3 **If N/PN to 3.2:** Could missingness in the  outcome depend on its true value? |  |  |  |  |  | NA / Y / PY / PN / N / NI |
| 3.4 If **Y/PY/NI to 3.3**: Is it likely that  missingness in the outcome depended on  its true value? |  |  |  |  |  | NA / Y / PY / PN / N / NI |
| Risk-of-bias judgement | Low | Low | Low | Low | Low | Low / High / Some concerns |
| Optional: What is the predicted direction of  bias due to missing outcome data? | NA | NA | NA | NA | NA | NA / Favours experimental /  Favours comparator /  Towards null /Away from  null / Unpredictable |

**Domain 4: Risk of bias in the measurement of the outcome**

| Signalling questions | (Wu et al., 2008) | (Rado and von Ammon Cavanaugh, 2016) | (Baptista et al., 2007) | (Baptista et al., 2006) | (Wang et al., 2020) | Response options |
| --- | --- | --- | --- | --- | --- | --- |
| 4.1 Was the method of measuring the  outcome inappropriate? | N | N | N | N | N | Y / PY / PN / N / NI |
| 4.2 Could measurement or ascertainment  of the outcome have differed between  intervention groups? | N | N | N | N | N | Y / PY / PN / N / NI |
| 4.3 If N/PN/NI to 4.1 and 4.2: Were  outcome assessors aware of the  intervention received by study  participants? | N | N | N | N | N | NA / Y / PY / PN / N / NI |
| 4.4 If Y/PY/NI to 4.3: Could assessment of  the outcome have been influenced by  knowledge of intervention received? |  |  |  |  |  | NA / Y / PY / PN / N / NI |
| 4.5 If Y/PY/NI to 4.4: Is it likely that  assessment of the outcome was influenced  by knowledge of intervention received? |  |  |  |  |  | NA / Y / PY / PN / N / NI |
| Risk-of-bias judgement | Low | Low | Low | Low | Low | Low / High / Some concerns |
| Optional: What is the predicted direction of  bias in measurement of the outcome? | NA | NA | NA | NA | NA | NA / Favours experimental /  Favours comparator /  Towards null /Away from  null / Unpredictable |

**Domain 5: Risk of bias in selection of the reported result**

| Signalling questions | (Wu et al., 2008) | (Rado and von Ammon Cavanaugh, 2016) | (Baptista et al., 2007) | (Baptista et al., 2006) | (Wang et al., 2020) | Response options |
| --- | --- | --- | --- | --- | --- | --- |
| 5.1 Were the data that produced this result  analysed in accordance with a pre-specified  analysis plan that was finalized before  unblinded outcome data were available for  analysis? | Y | NI | NI | NI | NI | Y / PY / PN / N / NI |
| Is the numerical result being assessed likely  to have been selected, on the basis of the  results, from... |  |  |  |  |  |  |
| 5.2. ... multiple eligible outcome  measurements (e.g. scales, definitions,  time points) within the outcome  domain? | N | N | N | N | N | Y / PY / PN / N / NI |
| 5.3 ... multiple eligible analyses of the  data? | N | N | N | N | N | Y / PY / PN / N / NI |
| Risk-of-bias judgement | Low | Low | Low | Low | Low | Low / High / Some concerns |
| Optional: What is the predicted direction of  bias due to selection of the reported result? | NA | NA | NA | NA | NA | NA / Favours experimental /  Favours comparator /  Towards null /Away from  null / Unpredictable |

**Overall risk of Bias**

|  | (Wu et al., 2008) | (Rado and von Ammon Cavanaugh, 2016) | (Baptista et al., 2007) | (Baptista et al., 2006) | (Wang et al., 2020) |  |
| --- | --- | --- | --- | --- | --- | --- |
| Risk of bias judgment | Low | Low | Low | Low | Low | Low / High /Some  concerns |
| Optional: What is the overall predicted  direction of bias for this outcome? | NA | NA | NA | NA | NA | NA / Favours  experimental / Favours  comparator / Towards  null /Away from null /  Unpredictable |

## ROBINS-I V2 tool [53]

| Domains Assessed | Risk of Bias outcomes(s) |
| --- | --- |
| Domain 1: Bias due to confounding | Low risk of bias except for concerns about uncontrolled confounding, Moderate risk, Serious risk, Critical |
| Domain 2: Risk of bias in classification of interventions | Low risk , Moderate risk of bias, Serious risk, Critical risk , |
| Domain 3: Bias in selection of participants in to the study | Low risk , Moderate risk , Serious risk , Critical risk |
| Domain 4: Bias due to deviations from intended interventions | Low risk, Moderate risk, Serious risk, Critical risk |
| Domain 5: Bias due to missing data | Low risk , moderate risk, serious risk ,critical risk |
| Domain 6: Bias in measure of the outcome | Low risk, moderate risk, critical risk |
| Domain 7: Bias in selection of the reported result | Low risk, Moderate risk, Serious risk, Critical risk |

….Continuation of Robins-I V2 tool. Judgment of bias based on domains

| Judgement | Interpretation | How reached |
| --- | --- | --- |
| *Low risk of bias except for concerns about uncontrolled confounding* | There is the possibility of uncontrolled confounding that has not been controlled for (given the observational nature of the study), but otherwise little or no concern about bias in the result | *Low risk of bias except for concerns about uncontrolled confounding* in Domain 1 and *Low risk of bias* in all other domains |
| *Moderate risk of bias* | There is some concern about bias in the result, although it is not clear that there is an important risk of bias | At least one domain is at *Moderate risk of bias*, but no domains are at *Serious risk of bias* or *Critical risk of bias* |
| *Serious risk of bias* | The study has some important problems: characteristics of the study give rise to a serious risk of bias in the result | At least one domain is at *Serious risk of bias*, but no domains are at *Critical risk of bias*  OR  Several domains are at *Moderate*, leading to an additive judgement of *Serious risk of bias* |
| *Critical risk of bias* | The study is very problematic: characteristics of the study give rise to a critical of bias in the result, such that the result should generally be excluded from evidence syntheses. | At least one domain is at *Critical risk of bias*  OR  Several domains are at *Serious risk of bias*, leading to an additive judgement of *Crticial risk of bias* |

Reference

1. Critical Appraisal Skills Programme, Systematic Reviews Checklist (CASP, n.d.), accessed January 13, 2025, .
2. J. A. C. Sterne, J. Savović, M. J. Page, et al., “RoB 2: A Revised Tool for Assessing Risk of Bias in Randomised Trials,” BMJ (2019): l4898, .
3. J. Sterne and J. Higgins, Risk of Bias Tools—ROBINS‐I V2 Tool 2024, accessed January 13, 2025, .
